# Cell polarity signalling at the birth of multicellularity: What can we learn from the first animals

**DOI:** 10.3389/fcell.2022.1024489

**Published:** 2022-11-24

**Authors:** Bree A. Wright, Marc Kvansakul, Bernd Schierwater, Patrick O. Humbert

**Affiliations:** ^1^ Department of Biochemistry and Chemistry, La Trobe Institute for Molecular Science, La Trobe University, Melbourne, VIC, Australia; ^2^ Research Centre for Molecular Cancer Prevention, La Trobe University, Melbourne, VIC, Australia; ^3^ Institute of Animal Ecology and Evolution, University of Veterinary Medicine Hannover, Foundation, Bünteweg, Hannover, Germany; ^4^ Department of Biochemistry and Pharmacology, University of Melbourne, Melbourne, VIC, Australia; ^5^ Department of Clinical Pathology, University of Melbourne, Melbourne, VIC, Australia

**Keywords:** cell polarity, basal metazoa, multicellularity, asymmetry, signalling, tissue architecture

## Abstract

The innovation of multicellularity has driven the unparalleled evolution of animals (Metazoa). But how is a multicellular organism formed and how is its architecture maintained faithfully? The defining properties and rules required for the establishment of the architecture of multicellular organisms include the development of adhesive cell interactions, orientation of division axis, and the ability to reposition daughter cells over long distances. Central to all these properties is the ability to generate asymmetry (polarity), coordinated by a highly conserved set of proteins known as cell polarity regulators. The cell polarity complexes, Scribble, Par and Crumbs, are considered to be a metazoan innovation with apicobasal polarity and adherens junctions both believed to be present in all animals. A better understanding of the fundamental mechanisms regulating cell polarity and tissue architecture should provide key insights into the development and regeneration of all animals including humans. Here we review what is currently known about cell polarity and its control in the most basal metazoans, and how these first examples of multicellular life can inform us about the core mechanisms of tissue organisation and repair, and ultimately diseases of tissue organisation, such as cancer.

## Introduction

Cell polarity refers to the intrinsic asymmetric distribution of macromolecules to distinct compartments of a cell to control directionality and coordinated polarisation. Cell polarity is associated with cell behaviours, such as migration and asymmetric cell division (Nelson 2003; Elsum et al., 2012; Butler and Wallingford 2017; Allam, Charnley, and Russell 2018; Stephens et al., 2018). The conservation through evolution of a vast majority of the cell polarity genes from basal metazoans to mammals highlights their significance and relevance in tissue architecture and cell behaviour (Goldstein and Macara 2007; Simons and Mlodzik 2008; Fahey and Degnan 2010; Elsum et al., 2012; Belahbib et al., 2018). Understanding cell polarity in basal metazoans may help unravel some of the mysteries of multicellularity and key processes that occurred during the transition from unicellular to multicellular organisms. The jump from unicellularity to multicellularity has occurred at least 25 times throughout evolution contributing to a complex tree of species, including: plants, fungi, amoeba and Bilateria to name a few (Grosberg and Strathmann 2007; Rokas 2008).

Advances in the understanding of the genetics of basal metazoans and unicellular organisms have provided opportunities to advance our understanding of signalling pathways that have been considered the main building blocks of multicellularity (Gerhart 1999). Here we extend this framework to include cell polarity signalling. Examination of multicellular events in unicellular organisms can shed light as to how the transition to multicellularity may have occurred and how early forms of cell polarity signalling may have enabled this. For example, Gram-negative bacteria *P. aeruginosa* demonstrates both kin selection (the progressive replication of a single cell to select for traits) (West et al., 2006) and cheating (the differential uptake of resources by certain cells, allowing for some cells to thrive at the cost of others) (Sandoz, Mitzimberg, and Schuster 2007; Dunny, Brickman, and Dworkin 2008). Another example is in the yeast species *S. cerevisiae* where cell polarity proteins *Sro7* and *Sro77*, (homologues of *D. melanogaster* Lgl (Lethal 2) giant larvae)), regulate polarisation of the actin cytoskeleton and vesicle exocytosis (Lehman et al., 1999; Gangar et al., 2005; Hattendorf et al., 2007). There are different hypotheses as to how multicellularity occurred (King 2004; Knoll 2011; Richter and King 2013; Sogabe et al., 2019) of which all fundamentally agree on the significance of cells orientating spatio-temporally to allow for coordinated cell movement, migration, and adhesion.

A key concept in the exploration of multicellularity is co-option–the ability for a trait to switch and thus impact on function (McLennan 2008). Evolutionarily, this occurs in many different contexts and here can be demonstrated by the co-option of genes already present in the genome being redirected to polarising events to support multicellularity. Cell adhesion molecules are considered one of these key co-optive processes (Abedin and King 2010; Harden, Wang, and Krieger 2016), another example being the LAP family of adaptor genes containing leucin rich repeats (LRR) and PDZ domains giving rise to the Scribble cell polarity gene (Santoni et al., 2002). Of note, the choanoflagellate genome (the closest unicellular organism relating to animals) reveals a rich repertoire of adhesion, cell polarity and signalling genes (King 2004; Snell et al., 2006; King et al., 2008).

In this review we will highlight our current knowledge of cell polarity in basal metazoans to further understand the evolution and adaptation of cell polarity signalling. It should be noted that there is still vigorous debate as to the evolutionary order of these lower metazoan animals as to how they relate to the last known common ancestor of bilaterians. However, this extends outside the scope of this review and we direct the reader to other references that tackle this important issue (see. (Grosberg and Strathmann 2007; Nosenko et al., 2013; Srivastava 2015; Schierwater et al., 2021a).).

## Transitioning to multicellularity: The first animals

Epithelial tissue is a key building block in the development of multicellularity due to the formation of epithelial sheets. ‘True’ epithelia is defined by 1) the presence of polarity between epithelial cells, 2) multiple junctions joining cells together, including: belt, septate, desmosome and tight junction, and 3) the presence of an extracellular matrix (Tyler 2003). The sheet formation acts as a barrier separating compartments of the organism, allowing for the regulation, diffusion and absorption of macromolecules (Tyler 2003; Fahey and Degnan 2012). To achieve such diverse functionality within an organism, epithelial cells need to be highly polarised, which is achieved by the asymmetric compartmentalising of cell polarity constituents (Rodriguez-Boulan and Nelson 1989; Elsum et al., 2012; Ebnet 2015; Wen and Zhang 2018). Basal metazoans are the first multicellular organisms and the ancient relatives to Bilateria, and more broadly the Eumetazoan subkingdom (Schierwater et al., 2021). They all contain examples of epithelial sheet formation, but only cnidarians have examples of true epithelia as explained above (Fahey and Degnan 2010; Rathbun, Everett, and Bergstralh 2022). The choanocytes in Poriferans (sponges) are considered epithelia-like (Simpson 1984) while the other epithelial cells lack key characteristics, like desmosomes and basal lamina (Fahey and Degnan 2010). Placozoans lack a basal lamina and key junctions associated with ‘true epithelia’. Although extracellular matrix (ECM) constituent genes such as *collagen*, *integrin-β* and *laminin*, are present and expressed in Placozoa. The absence of an actual ECM and basal lamina has been a peculiarity in the placozoans (Ringrose et al., 2013). These basal metazoans will be introduced briefly below.

### Placozoa

Phylum Placozoa comprises flat sea-dwelling animals approximately 1–5 mm in diameter and 20 μm in height. They are morphologically considered to be one of the simplest animals with no distinguishable organs, nerve or muscle cells, basal lamina or extracellular matrix (Smith et al., 2014; DuBuc, Ryan, and Martindale 2019; Schierwater et al., 2021). The most well-known species of placozoans is *Trichoplax adhaerens,* although a number of other species have been described and studied (Schierwater et al., 2021; Neumann et al., 2022). Structurally, placozoans consist of six different cell types, with 80% of the animal comprised of epithelial cells (Smith et al., 2014). Most essential signalling pathways are present in placozoans, including Wnt, Notch, cell adhesion molecules, mitogen-activated protein kinase (MAPK) signalling, NFκB and TGF-β at both the transcriptome and proteome level (Srivastava et al., 2008; Ringrose et al., 2013; Belahbib et al., 2018). Placozoans show a very high regenerative potential including the ability to re-aggregate animals from single cells (A. Ruthmann and Terwelp 1979; Osigus et al., 2022). No placozoan has been identified as having cancer, even when exposed to high levels of radiation (Fortunato et al., 2021).

### Porifera

Porifera, named due to their porous nature, encapsulates a diverse family of sponges. Their body plan consists of a labyrinth of small canals and chambers lined with choanocytes (cilia beating cells) that allows for the flow of water through the animal and the filtration of nutrients and microalgae (Soest et al., 2012). The well-studied marine Porifera *Amphimedon queenslandica* contains key regulatory, transcription, and signalling pathway genes including: Hox, Wnt, Hedgehog, TGF-ß, Notch, Jak/Stat, MAPK signalling pathway and cell adhesion molecules (Gerhart 1999; Nichols et al., 2006; Adamska et al., 2011; Wu et al., 2022). From a junctional perspective, Porifera have adherens junctions similar to those present in Bilateria, but there is no evidence of septate junctions or basal lamina (Srivastava et al., 2010). In the freshwater sponge *E. muelleri*, focal adhesion-like junctions and adherens junctions have been identified with highly conserved genes such as talin, integrin and focal adhesion kinase (Mitchell and Nichols 2019). Similar to other basal metazoans, sponges have the capacity to regenerate which has been reported to occur through the process of epithelial-to-mesenchymal transition (EMT) (Alexander et al., 2015; Lavrov et al., 2018; Ereskovsky et al., 2021; Wu et al., 2022).

### Ctenophora

Ctenophores more commonly known as comb jellies, consist of over 200 species and differ from other basal metazoans in that they have a characteristic set of eight comb rows that run along their length (Pang and Mark 2008; Tamm 2014). Morphologically, ctenophores consist of an epithelial ectoderm and endoderm with a mesoglea layer containing collagen filaments (Freeman 1977; Harrison and Ruppert 1991; Simmons, Pang, and Martindale 2012). Gene analysis of the ctenophore *M. leidyi* reveals a canonical Wnt signalling pathway similar to bilaterians (J. F. Ryan et al., 2013). However, Ctenophores lack key signalling and polarity genes like Scribble and crumbs (Belahbib et al., 2018) and members required for non-canonical Wnt signalling pathway (J. F. Ryan et al., 2013). Similar to placozoans, many ctenophore species examined do not contain a basal lamina (Ringrose et al., 2013). In the ctenophore *M. leidi*, key binding domains such as the groove-binding motif and cytoplasmic binding domain of E-Cadherin showed a lack of conservation compared with Placozoa, Porifera and Bilateria (Belahbib et al., 2018). The analysis of Ctenophores show a lack of gene conservation and it has been suggested this is due to secondary loss (Belahbib et al., 2018). One example is the lack of the MAGUK protein Dlg in ctenophores, which is a highly conserved gene present well before basal metazoans e.g. choanoflagellates (Fahey and Degnan 2010; Belahbib et al., 2018; Schiller and Bergstralh 2021).

### Cnidaria

Cnidarians encapsulate over 10,000 species that can be classified into two broad groups–sessile Anthozoa (e.g. the sea anemone *Nematostella vectensis*) and medusozoa (e.g. the freshwater *Hydra vulgaris*) (Technau and Steele 2011; Z.-Q. Zhang 2011). Similar to other basal metazoans, cnidarians have the capacity to regenerate lost or damaged body parts when both chemical or mechanical digestion occurs (P. M. Bode and Bode 1980; Layden, Rentzsch, and Röttinger 2016; Röttinger 2021). Studies of *Hydra* reveal the presence of ECM, cell-cell adhesion molecules, Wnt, hedgehog and notch signalling–which are all present and well conserved (Tucker and Adams 2014). A thorough review on the conservation of cell polarity signalling in Cnidaria has also recently been published (Rathbun, Everett, and Bergstralh 2022).

Core cell polarity signalling complexes in the basal metazoa. Several cell polarity signalling systems have developed through evolution, gaining complexity with evolving form and function of animal structures. In a few instances however, such as ctenophores or *C. elegans*, secondary loss of cell polarity genes have been observed (Belahbib et al., 2018). Analysis of genomic DNA sequences have identified the central cell polarity regulator complexes Scribble, Par and Crumbs in all basal metazoans (Srivastava et al., 2008; Fahey and Degnan 2010; Riesgo et al., 2014; Tucker and Adams 2014; Belahbib et al., 2018). These cell polarity signalling pathways remain fundamentally unexamined from a functional perspective in the basal metazoans. Here we seek to collate what is known of cell polarity signalling in basal metazoans, including the expression and function of cell polarity proteins, and to highlight the importance of these cell polarity mechanisms throughout evolution.

### The par, crumbs, and scribble modules in apico-basal polarity regulation

**FIGURE 1:**
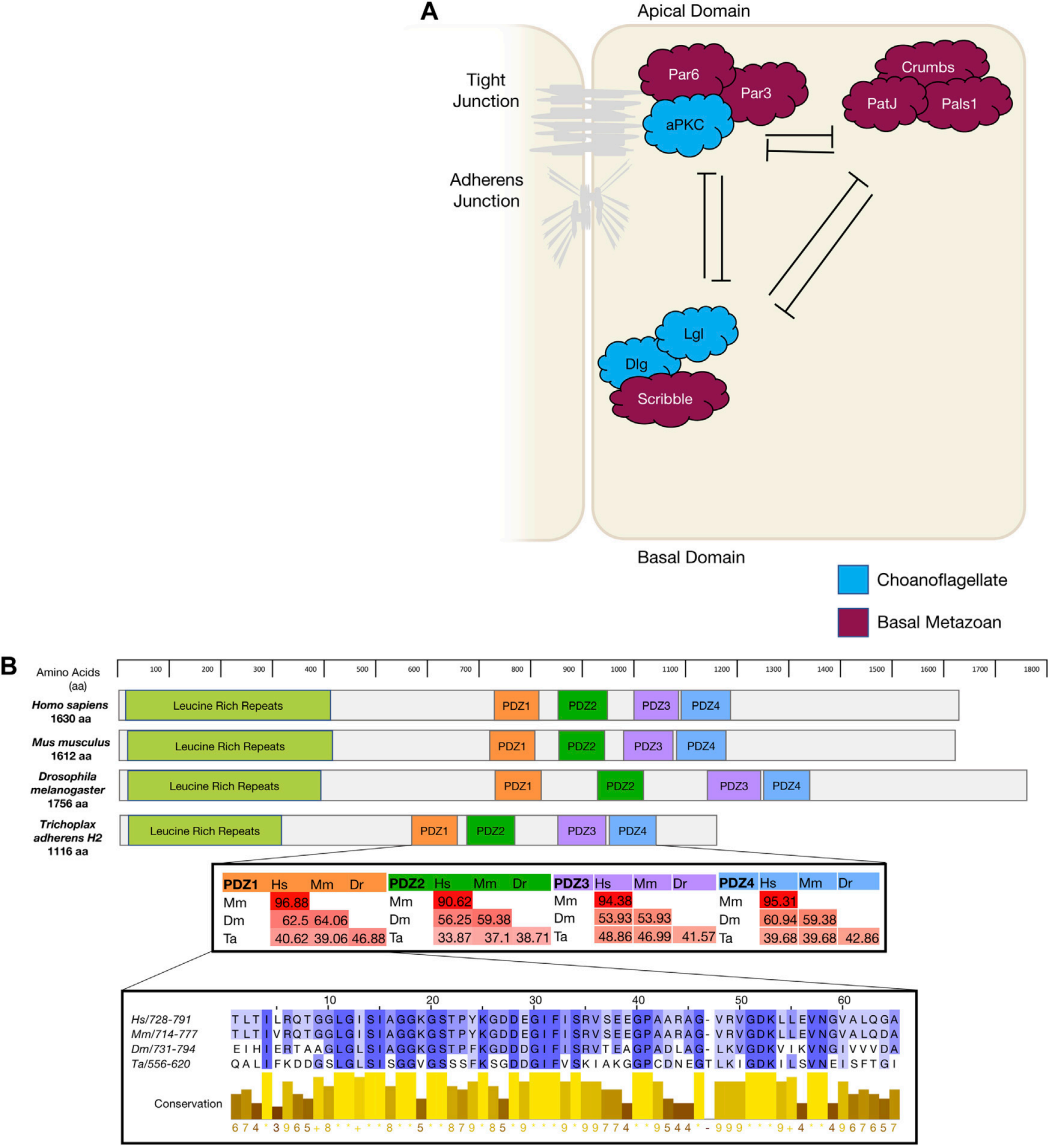
**(A)**. The diagram depicts mammalian apico-basal polarity proteins and their interactions and localisation within an epithelial cell and the appearance of these genes in evolution. **(B)**. Using Scribble as an example of gene conservation, a comparison has been made between the gene structure of four animals, a percentage map of key PDZ domains when compared between these four animals. PDZ1 has been expanded as an example of conservation and sequence similarity. Sequences were aligned in Clustal Omega and percentage conservation analysed in Jalview. Colour represented level of conservation. Sequences were sourced from uniport accessions: *H. sapiens* Q14160-1; *M. musculus* Q80U72-1; *D. melanogaster* Q7KRY7-1; and *T. adherens* A0A369S7Y8.

**Apico-basal polarity** is largely specific to epithelial cells and involves the localisation of polarity modules to the apical and basolateral membranes (Figure 1A) (Bilder and Perrimon 2000; Nelson 2003; Margolis and Borg 2005). Apico-basal polarity is considered essential in the formation of epithelial sheet and barrier formation, a concept fundamental to metazoan development. The polarising events of apico-basal localisation within a cell allow for formation of junctions between cells. Notably zonula adherens and tight junctions in vertebrates, adherens and septate junctions in *D. melanogaster* and apical junctions in *C. elegans* (Alberts et al., 2002; Knust and Bossinger 2002; Guillot and Lecuit 2013). Apico-basal polarity is associated with three modules: Crumbs, Par and Scribble, that were first identified in the model organisms *D. melanogaster and C. elegans* (Bilder and Perrimon 2000; Nelson 2003; Margolis and Borg 2005). The spatial localisation of these modules, along with their mutually antagonistic relationship, allows for the establishment of tissue architecture (Figure 1A). Further, it allows for proper epithelial movement, junctional cell interaction, substrate secretion, cell proliferation and apoptosis, and regulation of cell signalling (Elsum et al., 2012; Margolis and Borg 2005; U. Tepass et al., 2001; Nelson 2003; Stephens et al., 2018). Disruptions to these polarity modules have been linked to a loss of tissue architecture, loss of junctional integrity, mis-localisation of other polarity proteins and aberrant cell signalling that can lead to increased cell proliferation and cancer (Bilder 2004; Elsum et al., 2012; Gödde et al., 2014; Stephens et al., 2018).

**FIGURE 2:**
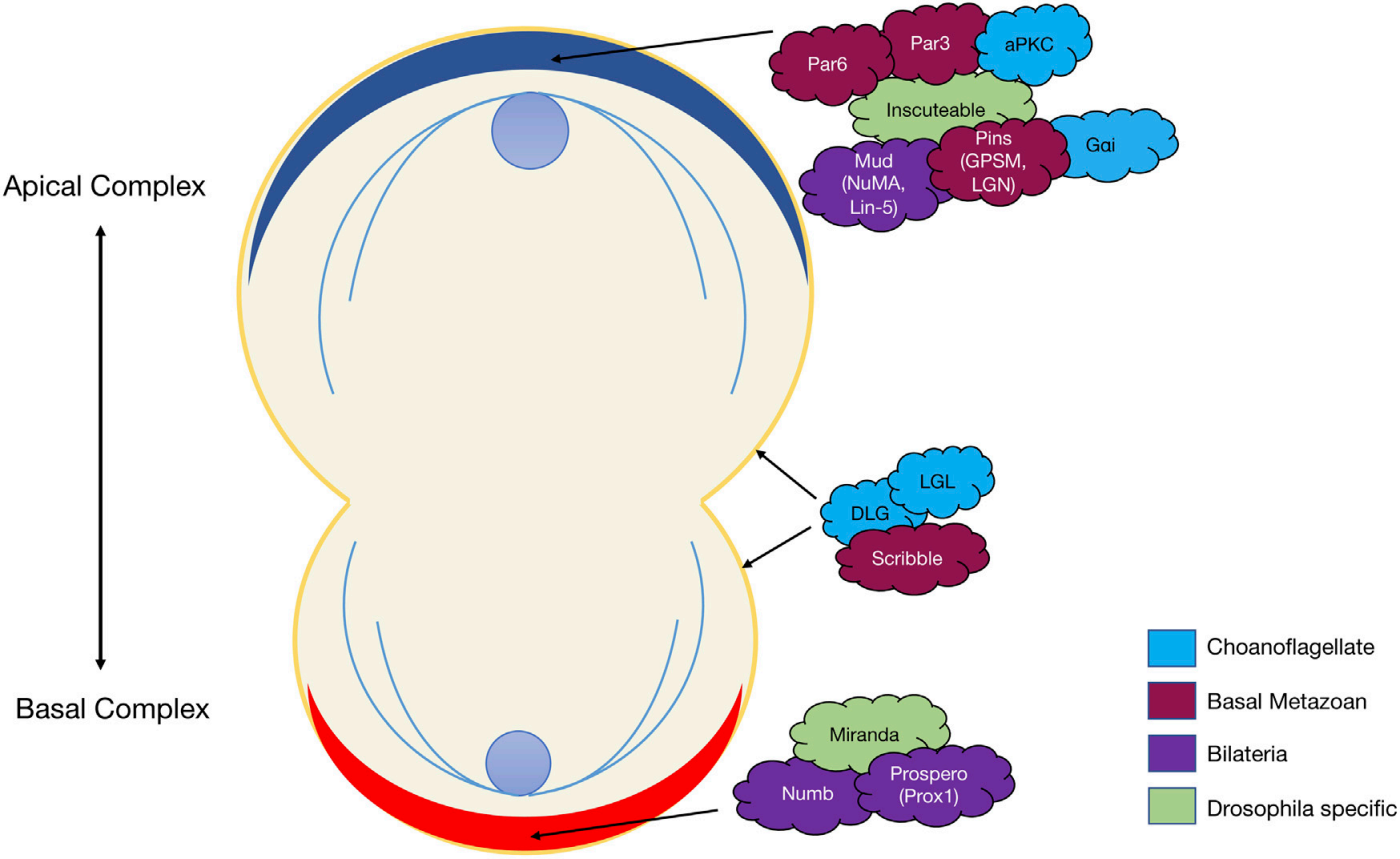
The diagram represents protein localisation in the *Drosophila* neuroblast as it undergoes asymmetric cell division. Colours represent the evolutionary appearance of these genes. Green proteins are *Drosophila* neuroblast ACD specific.

The par polarity complex first discovered in *C* *Elegans* (Kemphues et al., 1988) is considered to be a metazoan innovation (Fahey and Degnan 2010; Belahbib et al., 2018) and is responsible for the first asymmetric division in a zygote by establishing cortical polarity (Figure Figure1A and Figure 2) (Kemphues et al., 1988). The Par complex consists of scaffold proteins well known for their diverse roles in regulating cell polarity. In addition to asymmetric cell division, these proteins play an integral role in regulating many other polarity states including, apico-basal polarity, planar cell polarity and front-rear polarity (Petronczki and Knoblich 2001; Hurd et al., 2003; Goldstein and Macara 2007; Assemat et al., 2008; Etienne-Manneville 2008). The Par complex consists of three interacting proteins, Par3, Par6 and atypical protein kinase C (aPKC) that localise to junctional regions of epithelial cell. This allows for adherens junction and tight junction formation in vertebrates [Figure 1] (Matter and Balda 2003; St Johnston and Ahringer 2010; Wen and Zhang 2018).

Par complex genes have been identified in all the earliest basal metazoans and linked to a variety of polarity signalling contexts that co-evolved through evolution (Table 1) (Macara 2004; Magie and Martindale 2008; Belahbib et al., 2018). Indeed, this is illustrated by the strict evolutionary conservation of the interacting domains of Par proteins and the mechanisms regulating these interactions. For example, the lysine residue in PB1 (Phox and Bem1 binding module) domain of Par6 is responsible for the interaction between Par6 and aPKC, and the aPKC phosphorylation site (S/T) in Par3. This PBM domain remains highly conserved in all basal metazoans (Belahbib et al., 2018). Only a few functional experiments have been undertaken on the Par complex in basal metazoans. In the cnidarian *N. vectensis* functional investigation of the Par complex, a conserved role in maintaining cell-cell adhesion has been demonstrated. *N. vectensis* Par proteins (NvPar-3, NvPar-6, NvaPKC) were shown to localise within the cnidarian epithelium similarly to that seen in sheet epithelia of bilateria (Salinas-Saavedra et al., 2015; Salinas-Saavedra and Martindale, 2018). *N. vectensis* polyps expressing a dominant negative version of NvPar-3 showed leakage of fluorescent tracer dye demonstrating an ancestral role of the aPKC/Par complex in the maintenance of cell-cell adhesion and the paracellular boundary (SJs) of epithelial cells during animal development (Salinas-Saavedra and Martindale, 2018). Supporting this, knockout of *Nvpar-6* and *Nvpar-3* genes using CRISPR/Cas9 targeting resulted in loss of integrity of ectodermal epithelium including disruption of the cytoskeleton and adherens junctions (as visualised by ß-catenin localisation) (Salinas-Saavedra and Martindale, 2018). Clonal studies through single cell blastomere injections of CRISPR/Cas9 targeting *Nvpar-3* showed that the resulting clones of NvPar-3 knockout epithelial cells also lost their structural integrity inducing in this case cell extrusion, thus demonstrating a cell-autonomous role for the Par Complex in regulation of epithelial cell polarity (Salinas-Saavedra and Martindale, 2018). Studies such as these reinforce the notion that these newly established polarity systems in the early metazoans played a critical role in the establishment of multicellularity.

**TABLE 1:** Apico Basal Polarity proteins.

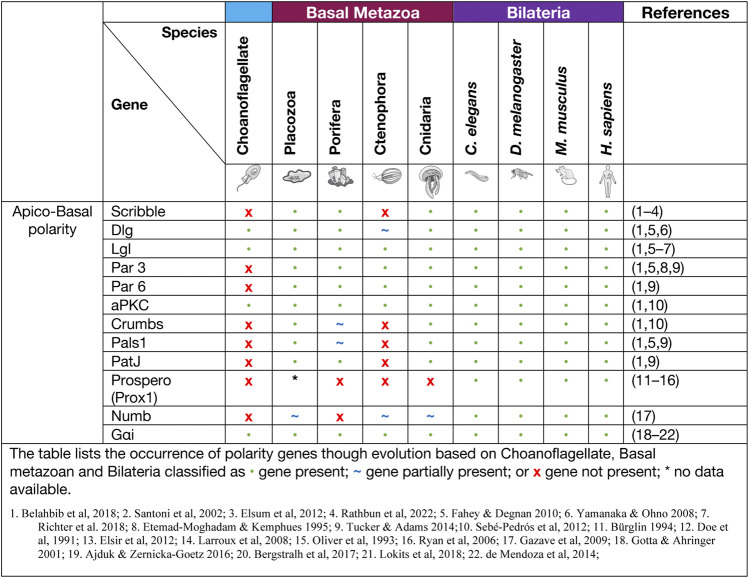

**The Crumbs polarity complex** is well documented as a critical complex in the development and stabilisation of apical adherent and tight junctions (Dow and Humbert 2007; Bazellieres et al., 2009; Bivic 2013; Ebnet 2015). The Crumbs complex consists of two scaffold proteins, Pals1 (Protein associated Lin seven 1) and PatJ (Pals1-associated tight junction), and a transmembrane protein Crumbs (Tepass, 2012). The Crumbs complex proteins are all metazoan developments and first appear in basal metazoans (Belahbib et al., 2018). Crumbs was first discovered in *D. melanogaster* (U. Tepass et al., 1990) and is the central molecule that acts as a scaffold for PatJ and Pals1 (Figure 1A). Genomic analysis revealed that the placozoan *T. adherens,* the cnidarian *N. vectensis, and the poriferan A. queenslandica* have conserved domains of Crumbs. Whereas the ctenophore *M. leidyi* most strikingly had no *crumbs* or *crumbs*-like gene that has been identified (Table 1) (Belahbib et al., 2018). Furthermore, analysis of the genomic DNA sequence of *A. queenslandica* revealed multiple *Crumbs*-like coding regions that are either variants of the gene, pseudogenes or truncated forms (Fahey and Degnan 2010). However, there are some questions as to the functional capacity of Crumbs in A. *queenslandica* (Fahey and Degnan 2010; Srivastava et al., 2010; Belahbib et al., 2018). Structurally, Crumbs has extracellular epidermal growth factor (EGF) domains interspersed with laminin repeats and a cytoplasmic tail consisting of two motifs; the FERM-binding motif (FBM) and a Class II PDZ protein binding domain (PBM) essential for the function of Crumbs proteins (Knust, Tepaß, and Wodarz 1993; Bivic 2013). Of note, the FBM domain responsible for aPKC binding in higher order species is depleted of two phosphorylation sites in *T. adherens* and in studied sponges (Belahbib et al., 2018).

Pals1 is a member of the Membrane-Associated Guanylate Kinase (MAGUK) family. The MAGUK family includes cell polarity genes that cumulatively are responsible for the organisation of protein complexes within a cell or at a cell or synaptic junction. Their localisation governs the polarisation of cells and their cytoskeleton filament connections (Mendoza et al., 2010). MAGUK genes extend past the metazoan lineage and have been identified in the choanoflagellate *M. brevicollis* and protist *C. owczarzaki* (Mendoza et al., 2010). The other members of the Crumbs complex Pals1 and PatJ have not been identified in ctenophores and their presence in sponges is unclear. In the sponge *A. queenslandica* a relative of Pals1 gene *MPP5/7*, that is also a member of the MAGUK family, is present and may play a substitutional role in the Crumbs complex (Fahey and Degnan 2010; Bivic 2013).

**The scribble polarity module** consists of a triad of scaffold proteins, Scribble, Lgl (Lethal giant larvae) and Dlg (Discs large), that localise to the basolateral membrane of epithelial cells (Figure 1A). The module has an important role in the control of tissue architecture and morphogenesis, and in tumour suppression (Bilder et al., 2000; Humbert, Russell, and Richardson 2003). Proteins of the Scribble module are major regulators of epithelial apico-basal polarity with broader roles in other forms of cell polarity (Stephens et al., 2018). Dlg and Lgl have been identified in lower order unicellular species (choanoflagellates and fungi), whereas Scribble is considered a metazoan innovation (Srivastava 2015; Belahbib et al., 2018). Scribble is a member of the LAP (Leucine rich repeat and Post-synaptic density-95/Discs-Large/Zo-1) family. Structurally, Scribble contains 16 Leucine rich repeats (LRR, a highly conserved protein motif that forms an arc-like structure), a LAP-specific domain (a domain related to LRR) and four PSD-95, ZO-1 and Discs large (PDZ) domains that coordinate the majority of Scribble’s binding interactions (Figure 1B) (Bilder and Perrimon 2000; Humbert, Russell, and Richardson 2003; Stephens et al., 2018; Bonello and Peifer 2019). A *Scribble* or *Scribble*-like gene has not been identified in unicellular organisms and does not appear to be present in the ctenophore *M. leidyi* (Belahbib et al., 2018). In ctenophores, it is thought to be due a secondary loss of the gene, and while no functional studies have been completed, it is postulated that the absence of a *Scribble* gene may result in variations of polarity complex localisation (Belahbib et al., 2018). As noted above, ctenophores also appear to lack a Dlg gene (Schiller and Bergstralh 2021). Analysis of different porifera classes identified key polarity proteins, including Scribble, Lgl and Dlg, responsible for cell adhesion and epithelial development (Fahey and Degnan 2010; Riesgo et al., 2014). Dlg from a structural perspective, contains three PDZ domains, SH3 domain and a GUK domain. As a scaffold protein, Dlg is part of the Post-Synaptic Density (PSD) family. This family is responsible for the maintenance, anchorage and structural localisation of other PSD structures and proteins in relation to neurotransmitter receptors and signalling channels (Sakarya et al., 2007; Alié and Manuel 2010). In metazoan species that do not contain nerve structures, such as Placozoans and Porifera, it was found that these PSD proteins were present and contained near identical interacting domains when compared to their mammalian counterparts. Furthermore, it is suggested that Dlg and other PSD genes like Homer (scaffold protein involved in Ca^2+^ signalling and transport) may play significant roles in these metazoan species as Ca^2+^ receptors and signalling communicators (Alié and Manuel 2010). The significance of PSD proteins, specifically Dlg, is highlighted by the full conservation of their residues that interact with PDZ domains compared with their human orthologues (Sakarya et al., 2007). Of note, imaging of Placozoan epithelium using staining with a pan-human Dlg antibody show an identical basolateral cortical staining to that seen for Dlg in Bilateria epithelium suggesting that TaDlg may have a conserved function in the regulation of Trichoplax epithelium (Smith et al., 2014). Lgl is the most ancient gene with homologues found in yeast (Sro7 and Sro77) where it regulates polarised exocytosis (X. Zhang et al., 2005; Grosshans et al., 2006; Müsch et al., 2002). This has been similarly compared to mammalian Lgl in basolateral exocytosis (Müsch et al., 2002). High levels of conservation of polarity genes from the Scribble, Par and Crumbs complexes have been identified in cnidarians when compared to bilateria (Rathbun, Everett, and Bergstralh 2022).

### Planar cell polarity signalling

**FIGURE 3:**
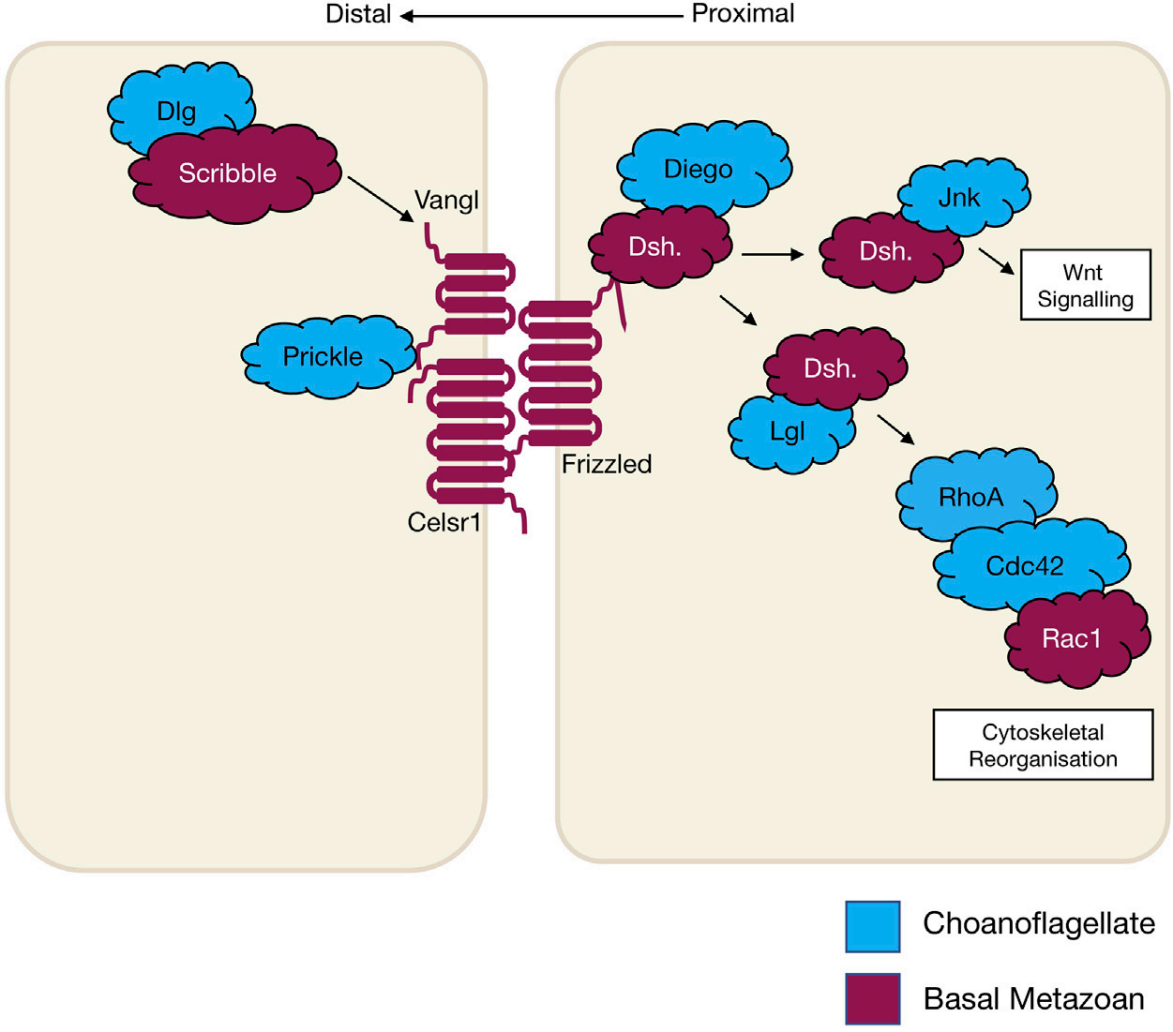
The schematic diagram shows core planar cell polarity pathways and their relationship to Wnt signalling and cytoskeletal reorganisation that occurs in wound healing. The different colours represent the evolutionary appearance of these genes.

Planar cell polarity (PCP), also referred to as tissue polarity or the non-canonical Wnt signalling pathway, is the global organisation of cells along a x/*y* axis in a plane (Figure 3). PCP signalling is essential for normal tissue development, cell homeostasis, axis determination and tissue morphogenesis (Simons and Mlodzik 2008; Butler and Wallingford 2017). Junctional PCP genes were first identified and have been extensively studied in the fly *D. melanogaster* (Gubb and García-Bellido 1982; Axelrod 2001; Adler 2002; Hale and Strutt 2015). The organisation of six transmembrane proteins on opposing sides of a cell allow for communication and coordinated interactions, including polarising events. The polarising events allow for the asymmetric placement of cilia or hairs and the orientation of the mitotic spindle (Goodrich and David 2011; Schenkelaars et al., 2016; Butler and Wallingford 2017). Downstream from the core PCP signalling, the PCP protein Dishevelled interacts with Lgl, Cdc42, RhoA and Rac1. These interactions aid in cytoskeleton re-organisation, maintaining adherens junctions, and when interacting with Jnk, feeds into Wnt signalling pathway (Figure 3) (Milgrom-Hoffman and Humbert 2018; Wiese, Nusse, and van Amerongen 2018). On examination of PCP signalling, Frizzled, Dishevelled and Prickle have all been identified in the four basal metazoans, whereas Celsr1 (Flamingo) and Vangl (Strabismus) are not found in ctenophores, nor Inversin (Diego) in porifera (Table 2) (Adamska et al., 2010; Srivastava et al., 2008; Schenkelaars et al., 2016; Belahbib et al., 2018; J. F. Ryan et al., 2013; Momose, Kraus, and Houliston 2012). Phylogenetic analysis of *prickle* and *prickle*-like genes reveals an ancestor of the gene in choanoflagellates. In the basal metazoans placozoa and cnidaria, it diverges from one to two genes–*prickle* and *testin* (Schenkelaars et al., 2016).

**TABLE 2:** Planar Cell Polarity proteins.

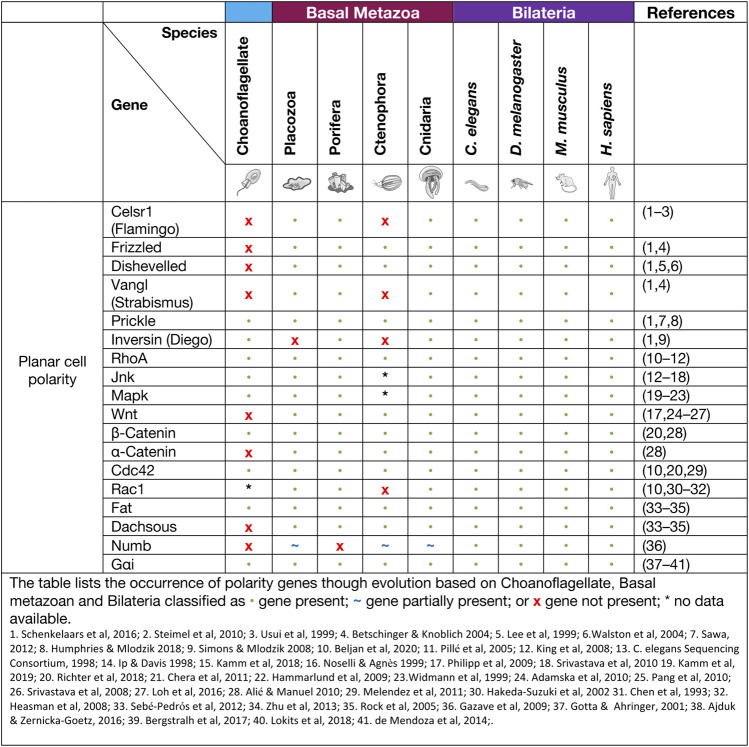

Examination of the cnidarian *C. hemisphaerica* larva stages reveal established PCP characteristics of oral-arboreal polarity and the formation of directionally organised cilium in each epithelial cell similar to that described in bilaterians (Momose, Kraus, and Houliston 2012; Milgrom-Hoffman and Humbert 2018). Further, *vangl* mRNA expression levels were evident throughout embryogenesis, elongation and ciliogenesis with enrichment occurring to the axis of the developing hydrozoan (Momose, Kraus, and Houliston 2012). When *vangl* was knocked down in the cnidarian *N. vectensis,* the embryos failed to undergo gastrulation or primary invagination, however this did not impact ß-catenin nuclear localisation, which in bilaterians is tightly coupled. Thus cell fate specification of the endoderm may have developed separately to other PCP/Wnt signalling pathways (Kumburegama et al., 2011).

Non-canonical Wnt signalling investigations in the cnidarian *Hydra* revealed specific Wnt pathway genes (*wnt5*, *wnt8*, *frizzled* and *dishevelled*) are all required for correct evagination of the bud and tentacle of the *Hydra*. The upregulation of these genes can be correlated to the activation of Wnt/ß-catenin signalling during tissue morphogenesis and development of the *Hydra* pulp (Philipp et al., 2009). Planar cell polarity genes *fat* and *fat*-like genes are associated with cell directional migration and morphogenesis in asymmetric cell division in bilaterians (Matis and Axelrod 2013). The *Hydra fat* and *dachsous* genes localise to the body of the animals where continuous growth and migration of cells occur supporting the theory of a similar role to that of bilaterians (Brooun et al., 2019). Phylogenetic examination of *frizzled* in a variety of different poriferan, placozoan, cnidarian and ctenophore species show multiple orthologues of *frizzled.* In some porifera and cnidarians, up to four *frizzled* orthologues have been identified, with evidence that the vertebrate paralogue of *frizzled* is an amalgamation of ancestral *frizzled* genes (Schenkelaars et al., 2015). The genes *flamingo, inversin* and *vangl* are PCP genes considered to be secondarily lost from the ctenophore *M. leidyi* (Table. 2) (J. F. Ryan et al., 2013; Schenkelaars et al., 2016), whereas, *dishevelled*, *frizzled* and *prickle* are present in all metazoans (Srivastava et al., 2008; Schenkelaars et al., 2016). Functional studies relating to specific pathway significance between basal metazoan PCP signalling and its similarities or differences to higher order species are ongoing.

### Asymmetric cell division signalling

Asymmetric cell division (ACD) refers to the specific localisation of cell fate determinants during cell division to establish two different cell characteristics (mother/daughter) (Knoblich 2001). In early cell division in the model organism *D. melanogaster,* asymmetric molecules Par3, Par6, aPKC, Inscuteable, Pins, Gαi and Mud localise to the apical cortex of the mitotic spindle, while cell fate factors Numb, Brat, Prospero, Pon and Miranda localise to the basal cortex (Figure 2) (Boyd et al., 1996; Tabuse et al., 1998; Hung and Kemphues 1999; Joberty et al., 2000; Kelsom and Lu 2012). Additionally, in *Drosophila* neuroblasts, the Scribble module proteins Scribble, Dlg and Lgl are important in ACD where they assist in mitotic spindle orientation (Elsum et al., 2012). The asymmetric localisation of these key polarity genes induces separation of the cells in an asymmetric fashion and therefore diversification of tissue types. A failure for polarity proteins to localise to the poles of the mitotic spindle is associated with defects in basal protein targeting, symmetric division, reduced spindle size or inverted neuroblast cell division (Bilder and Perrimon 2000; Albertson and Doe 2003; Neumüller and Knoblich 2009; Royer and Lu 2011). The diversification of ACD has been identified in prokaryote and eukaryotic organisms, basal metazoans and bilaterians (Table 3) (K. R. Ryan and Shapiro 2003; Knoblich 2001). It should be noted that cells at an early embryonic stage have the capacity to divide either asymmetrically, as described above, or symmetrically where two identical daughter cells are formed (Knoblich 2001; Schenkelaars et al., 2017). The selective differential distribution of protein and RNA into daughter cells is the foundation for the development of different tissue or cell types within an organism, referred to as cell fate (Jan and January 1998; Knoblich 2010).

**TABLE 3:** Asymmetric Cell Division proteins.

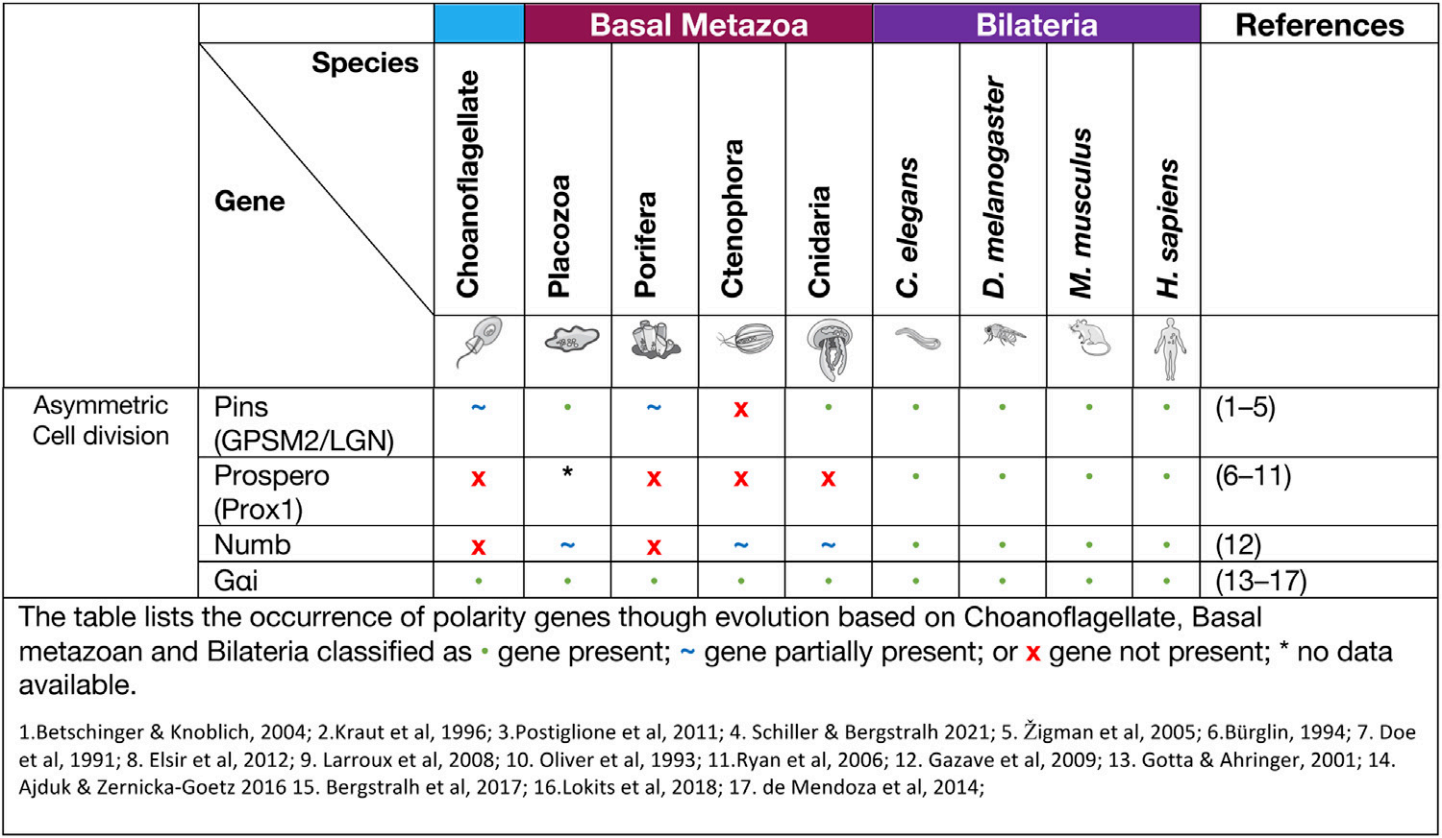

Asymmetric cell division allows for the development of both germ cells and somatic cells that form different cell lineages and allow for plasticity of the cells in processes, such as reaggregation. In the cnidarian *H. vulgaris,* multipotent interstitial cells have the capacity to differentiate into gametes and almost all somatic cell lines (Bosch 2004; H. R. Bode 1996; Bosch and David 1987). The pliability of cnidarian cells and their capacity to adapt to their environment is remarkable, with the examples of an adult medusa metamorphosis into a polyp (Piraino et al., 1996). Another example is the cnidarian *Podocoryne carnea* that through the process of asymmetric cell division can differentiate medusae formed cells into an unrelated phenotype e.g. Muscle cells to nerve cells (Schmid and Alder 1984; Seipel, Yanze, and Schmid 2003). One of the proteins associated with ACD is Pins (also known as LGN or GPSM2). Pins has been shown to interact closely with Dlg in spindle orientation and this interaction is believed to have evolved in cnidarians (Schiller and Bergstralh 2021). The placozoan *T. adhaerens* and the sponge *A. queenslandica* do not contain the key linker regions required for GPSM2 to interact with Dlg, however they do contain other key motifs of GPSM2. It is postulated that these conserved regions may still be able to play a part in ACD, cell orientation and division (Schiller and Bergstralh 2021). In Porifera, during initial embryonic development, asymmetric division of macromeres to micromeres occur while later in embryonic development there is more evidence for higher levels of symmetric cell divisions. In the freshwater sponge *E. fluviatilis* the paralogue gene *Musashi* (a gene required for stem cell maintenance in *Drosophila*) has been identified as being specifically expressed in stem cells and regulates sustainable regeneration. This is the earliest occurrence of this gene in basal metazoans and of its role in ACD (Okamoto et al., 2012).

## Cell junction complexes in the basal metazoa

### Adherens junctions

**FIGURE 4:**
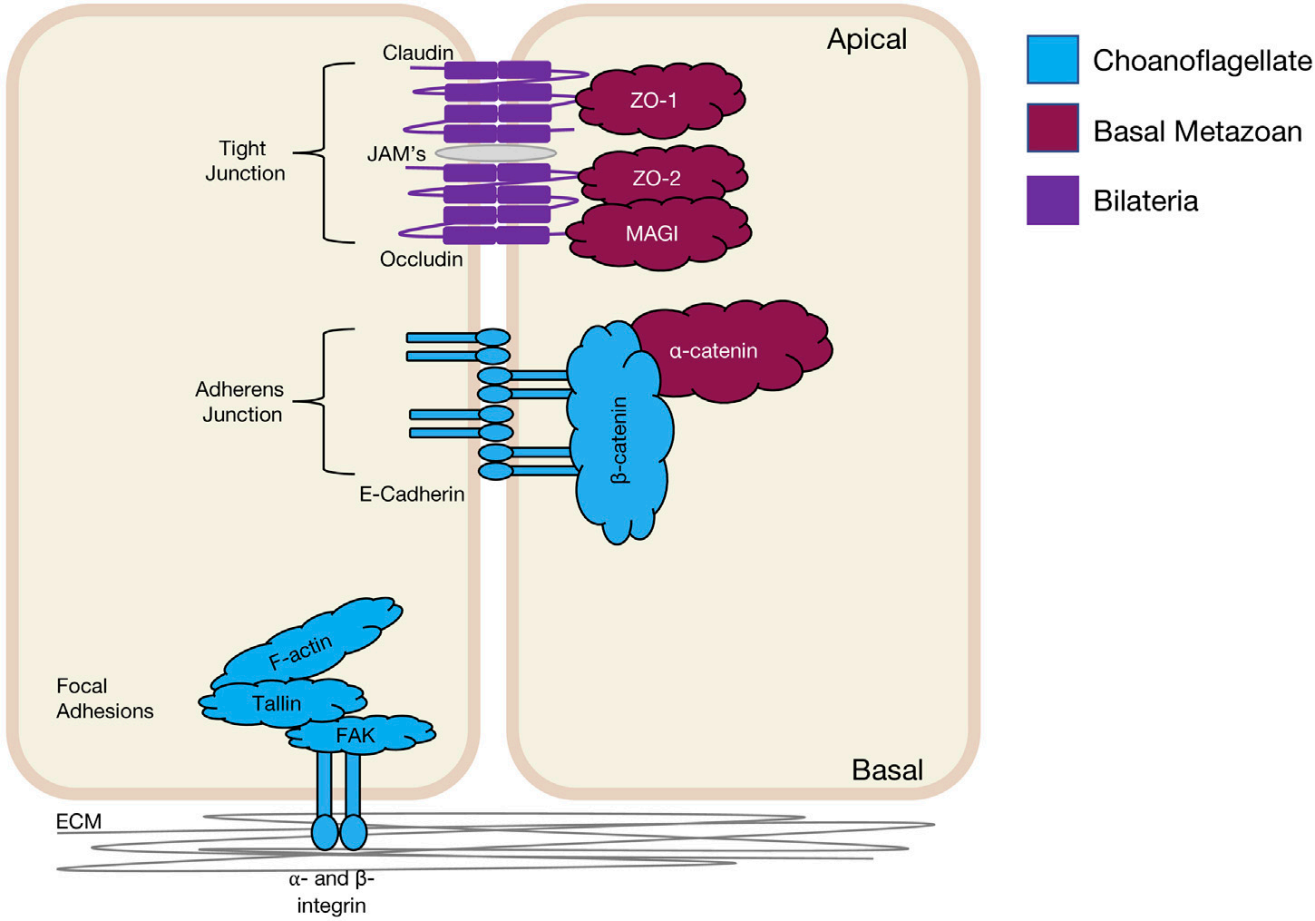
Bilaterian representation of epithelial cell-cell and cell-ECM junctions and their emergence in evolution. It should be noted that for Claudin, whilst represented as a basal metazoan innovation, it has only been identified in Cnidaria. There are Occludin-like genes present in basal metazoans but it is not known if they have the same functional properties as in Bilateria.

Adherens junctions, also known as Zonula Adherens, form belt-like junctions that act as a conduit between the apical and basal domains of epithelial cells (Figure 4). Adherens junctions are acknowledged as the most common junction in animal epithelia (Oda and Takeichi 2011; Hiroki 2012). Adherens junctions have been identified in placozoans, cnidarians and ctenophores with none so far identified in Porifera (table. 4) (T. J. C. Harris and Ulrich 2010; Salinas-Saavedra and Martindale, 2019). The presence of adherens junctions in placozoans appears crucial for their tissue integrity as no other junctions have been identified placozoans to date (Smith and Reese 2016).

**TABLE 4:** Junctional proteins.

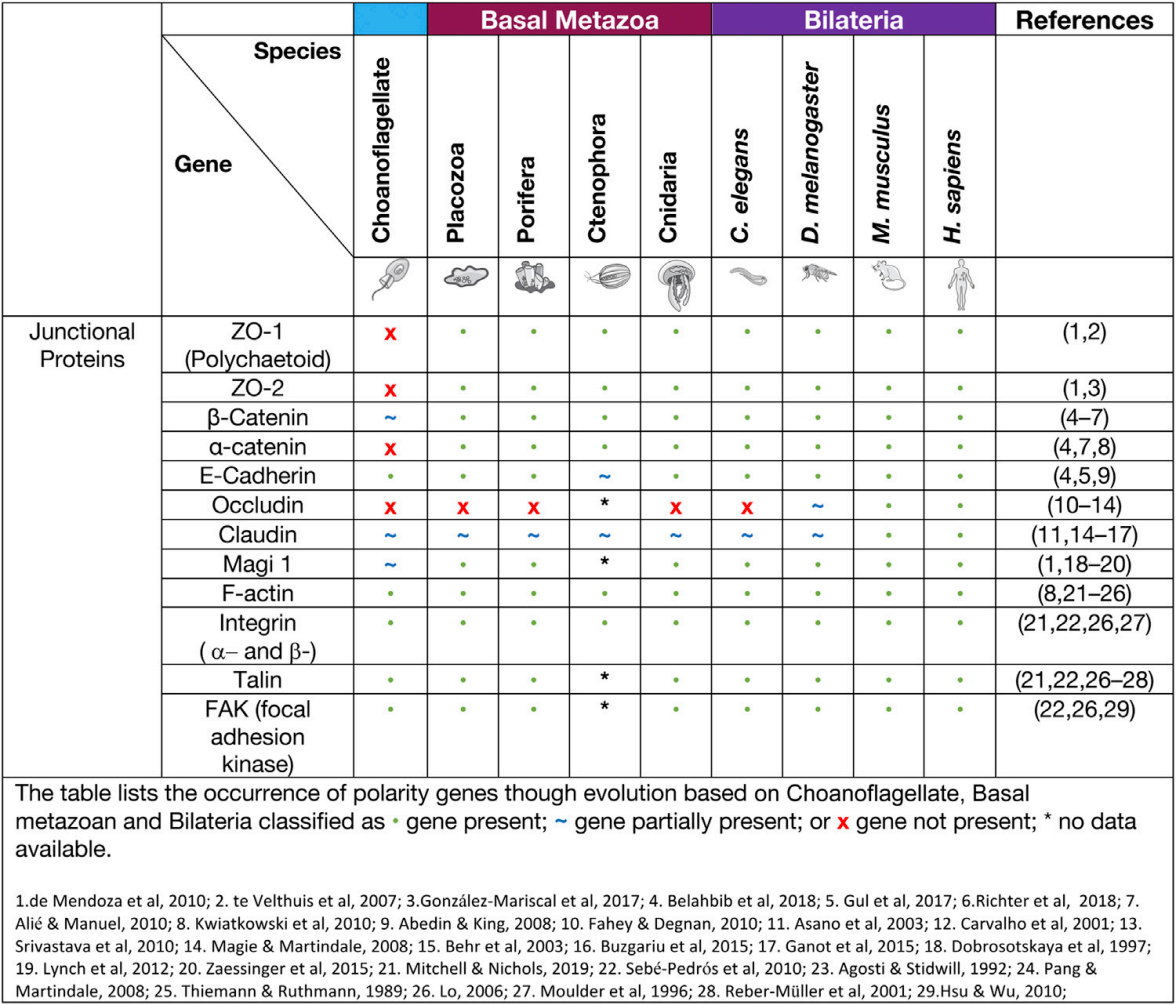

### Cadherin-catenin complexes

A major component of adherens junctions are cadherin-catenin complexes. Classical cadherins date back to the Urmetazoan (the hypothetical last common ancestor of all animals or metazoans) and are type I transmembrane proteins that consist of calcium-dependent transmembrane cell adhesion molecules (CAMs) that form adherens junctions associated with cell-cell adhesion, embryonic development, and cell morphogenesis (Hulpiau and van Roy 2011; T. J. C. Harris and Ulrich 2010; King, Hittinger, and Carroll 2003; Gul et al., 2017). The cadherins are a superfamily of proteins containing at least two cadherin repeats and can be classified into three families: Major cadherins, protocadherins and cadherin-related genes (Gul et al., 2017). Placozoans contain cadherin and cadherin-related genes, whereas cnidaria contain multiple genes of all three cadherin families (Gul et al., 2017; Hulpiau, Gul, and van Roy 2013; S. A. Nichols et al., 2012). When examining the current literature of cadherins in the basal metazoans it was found that placozoans, poriferans and cnidarians all have identifiable E-cadherin with necessary binding motifs (Table 4). *M. leidyi* (Ctenophora) has E-cadherin motifs but show high levels of divergence that raises doubt to its capacity to bind to known interacting genes such as ß-catenin and p120 (Belahbib et al., 2018; Hulpiau and van Roy 2011; S. A. Nichols et al., 2012; Ringrose et al., 2013; Srivastava et al., 2008).

Catenins that form part of adherens junctions can be placed into three sub-families: p120-, α- and β-, with examples of each subfamily identified in the basal metazoans, with β-catenin being identified in many unicellular organisms (Alié and Manuel 2010; Belahbib et al., 2018). Catenins, including α- and β-catenin bind filamentous actin (F-actin) within the cell and cadherins within the adherens junctions to form a semi-permeable barrier between anterior and posterior of the cell (Baum and Georgiou 2011; Gooding, Yap, and Ikura 2004; Tian et al., 2011; Nelson 2008; T. J. C. Harris and Ulrich 2010; Magie and Martindale 2008). A major contributor to adherens junction homeostasis is the presence of β-catenin in higher order metazoans and some basal metazoans. In the ctenophore *M. leidyi,* β-catenin does not localise to the cell junctions most likely due to the lack of a cytoplasmic domain essential for β-catenin binding. It was further concluded that this may indicate that the ancestral role of β-catenin was in cell-fate specification associated with Lef/Tcf co-factors that enter the nucleus to regulate canonical Wnt signalling rather than cell adhesion (Salinas-Saavedra and Martindale, 2019). The porifera *A. queenslandic*a contain junctional proteins cadherin1 and α-catenin1-like gene, however within the middle of the gene a stretch sequence has been identified that is not otherwise seen in bilaterian counterparts (Fahey and Degnan 2010). The evidence is still lacking regarding other regulators of adherens junctions, except for the Par complex as discussed previously. Indeed, adherens junctions in the cnidarian *N. vectensis* ectodermal epithelial cells are responsible for the localisation of the Par complex and if disrupted a loss of integrity and loss of solute permeability has been observed (Salinas-Saavedra and Martindale, 2018).

### Tight and septate junctions

Tight junctions are attributed to vertebrate species and act as a junctional barrier regulating the diffusion of macromolecules between and through cells (Matter and Balda 2003). Located at the apical region of cells, tight junctions consist of transmembrane signalling proteins, such as Claudin, Occludin, junctional adhesion molecules (JAMs), and adaptor proteins, such as ZO (Zonula Occludin) -1, -2, -3, polarity proteins Par -3, -6, Pals1, PatJ and Magi -1,-2 and -3 (Figure 4) (Fanning et al., 1998; Tsukita, Furuse, and Itoh 2001; Matter and Balda 2003; Niessen 2007; Steed, Balda, and Matter 2010; Hartmann et al., 2020). ZO-1 as a member of the MAGUK family is responsible for junctional organisation and regulation of proteins, such as ZO-2, Occludin and F-actin. These interactions allow linking and binding to the cortical actin cytoskeleton of the cell (Fanning et al., 1998; Itoh et al., 1999). The ZO proteins have been identified in all four basal metazoan lineages (Table 4) (Mendoza et al., 2010). Interestingly, electron microscopy studies have failed to reveal tight junctions in the placozoan *T. adherens*. This is a peculiarity as the genome contains *Z O -1* and *Claudins* that are associated with tight junctions (Mendoza et al., 2010; González-Mariscal et al., 2017; Belahbib et al., 2018). Although tight junctions are ‘stricto-sensu’ vertebrate specific, genes associated with tight junctions have been identified in invertebrates, basal metazoans and choanoflagellates and hence referred to as ‘claudin-like’ (Ganot et al., 2015). For example, In the cnidarian *Hydra,* 14 claudin-like genes have been identified, with 10 of them specifically in the ectoderm and/or endoderm (Buzgariu et al., 2015) and claudin-like genes in *Drosophila* have been associated with septate junctions (Behr, Riedel, and Schuh 2003). Septate junctions are cell-cell junctions that appear ladder-like under electron microscope and aid in solute diffusion and structural support (Matter and Balda 2003). Septate junctions have been identified in *Hydra*, containing a ladder-like structure that in reaggregation studies forms within hours (Filshie and Flower 1977; Seybold, Salvenmoser, and Hobmayer 2016). A similar structure has also been noted in *Trichoplax* in the proximal cells of the animal that appear ‘ladder-like’ but are periodic in nature. No such junctions have been identified in porifera (Ruthmann et al., 1986; Ganot et al., 2015). Cnidarians display both the required genes and structure to form septate junctions similar to those found in Bilateria (Ganot et al., 2015; Rathbun, Everett, and Bergstralh 2022). This is not seen in ctenophores, where only claudin-like genes have been identified (Ganot et al., 2015).

### Focal adhesions and integrin complexes

Focal adhesions are protein-rich structures where the integrin transmembrane proteins provide adhesion between cells and the extracellular matrix (ECM) (Figure 4). Like cadherins, integrins also represent signalling hubs (Michael and Parsons 2020). Focal adhesion genes surpass the age of the earliest metazoan lineages, believed to stretch back into the Cambrian time (S. A. Nichols et al., 2012). Whereas, components of the integrin machinery predate the metazoan lineage (Sebé-Pedrós et al., 2010). Integrin receptors that are composed of membrane-anchored heterodimer receptors have been reported in species of marine sponges (Müller 2003). More recently, the focal adhesion proteins, integrin, talin, and focal adhesion kinase (FAK), have been shown to form a complex that localises to the cell-cell junctions and extracellular matrix adhesions in the freshwater sponge *E. mulleri* (Mitchell and Nichols 2019). Of note, focal adhesion associated molecules integrin, vinculin, paxillin, talin and FAK are all found and expressed in *Trichoplax* (Srivastava et al., 2008), although a basement membrane structure does not appear present in these animals suggesting either a secondary loss in Placozoa or an independent gain in the other basal metazoans (Fidler et al., 2017).

## Cell polarity in the basal metazoa and the origin of multicellularity

Here we have reviewed key signalling pathways regulating cell polarity and adhesion in the basal metazoan species and how this relates to the evolution of complex tissues such as epithelial structures. The examination of such pathways not only gives us knowledge into the ancient function of these genes and when they arose, but allows us to examine their role in the advent of multicellularity. A key challenge for a multicellular organism is to organise tissue architecture to drive cellular and organismic function. For the evolution of a multicellular animal to occur, a number of events are required including the development of cell differentiation and adhesive cell interactions within the epithelium, the orientation of division axis, and the ability to reposition daughter cells over long distances so as to establish and maintain a body plan. In addition, to obtain the division of labour that is linked with multicellularity, the process of differentiation that generates various cell types must be properly controlled. Asymmetry and cell polarity provides a universal tool for building multicellular tissue architecture. Although asymmetry can occur by stochastic means, extrinsic cues whether chemical or mechanical are more reliable and provide robustness to generate the asymmetry required for tissue architecture. Cell polarity and cell adhesion mechanisms relay these external cues internally to re-organise cell and tissue as well as provide a link with transcriptional programs required for tissue morphogenesis.

Apico-basal cell polarity mechanisms first appear in basal metazoans, and based on the simultaneous presentation of multicellularity and these cell polarity constituents, it is reasonable to propose that cell polarity mechanisms played a key role in this process. As discussed, other cell polarity genes are far more ancient and extend back into unicellular organisms. For example, β-catenin’s ancestral function appears related to TCF/LEF transcriptional regulation of Wnt signalling rather than junctional polarity and thus provides another example of co-option in cell polarity systems linking nuclear transcriptional programs to newly minted cell adhesion mechanisms (Salinas-Saavedra and Martindale, 2019). Interestingly, of the basal metazoans, ctenophores appear to be outliers at this point in terms of cell polarity mechanisms. The significant cell polarity associated gene loss in ctenophores raises interesting questions as to the alternative mechanisms by which ctenophores control various aspects of cell polarity, tissue organisation and its repair. In addition, the potential role of tight junction proteins in basal metazoans is an interesting enigma and could provide new insights into the evolution of these permeability barriers. As discussed, many basal metazoans produce tight-junction proteins (e.g. *Zo-1*) despite the absence of tight or septate junctions. This may indicate a more ancient divergent function for these genes that has been co-opted for the regulation of tight junctions. Interestingly, Polychaetoid, the *Drosophila* ZO-1 homologue, localises to adherens junctions and provides a link to actin regulation (Takahashi et al., 1998; Wei and Ellis 2001; Choi et al., 2011), pointing to a possible similar role for ZO-1 in basal organisms.

Research in the basal metazoans provides the opportunity to understand the fundamental building blocks of multicellularity and by extension its relationship to key events such as cancer. Indeed, examination of the evolutionary origin of cancer-related protein domains suggests two peaks, one at the time of the origin of the first cell and the other around the time of the evolution of the first multicellular organisms (Domazet-Lošo and Tautz 2010). Importantly, this second peak dubbed “gate-keeper” genes consist of oncogenes and tumour suppressors whose mutations promote tumour progression through altering cell proliferation, inhibiting differentiation or inhibiting cell death. This second peak also corresponds to the advent of the cell polarity signalling pathways in early basal metazoans described in this review. As many of these cell polarity regulators have been linked to tumour suppression in Bilateria (Stephens et al., 2018), examining the mechanisms of cell polarity and tissue architecture regulation in basal metazoans is likely to lead to fundamental insights into the origins of cancer. Almost all bilaterian animals have reported examples of cancer formation (Aktipis et al., 2015). Indeed, sponges and *Hydra* have reported cases of cancer that mimic that of higher order species, such as intrusive proliferation, loss of tissue architecture and a loss of specialised tissue (Hanahan and Weinberg 2000; Aktipis et al., 2015). The advent of multicellularity required new molecular mechanisms that allowed cellular cooperation and suppressed any cellular conflicts that enhance individual cell fitness to the detriment of the organism (Aktipis et al., 2015; Madan, Gogna, and Moreno 2018; Bowling, Lawlor, and Rodríguez 2019). From this point of view, cancer would represent a breakdown of this multicellular cooperation with over-competitive cells effectively “cheating”, leading to overall loss of fitness of the organism (Rainey 2007; Aktipis et al., 2015). Importantly, cell polarity and tissue architecture regulators play key roles in regulation of cell competition mechanism in Bilateria (Madan, Gogna, and Moreno 2018; Bowling, Lawlor, and Rodríguez 2019; Fahey-Lozano et al., 2019; Baker 2020). We therefore contend that cell competition mechanisms first appeared in basal metazoans and are mechanistically linked to the acquisition of the original cell polarity mechanisms required for the advent of multicellularity. The ability to generate tissue chimaeras in basal metazoans such as *Trichoplax* and *Hydra* (Klimovich, Wittlieb, and Bosch 2019; Schierwater et al., 2021) provides an attractive system to explore how cell competition mechanisms may have first appeared in basal metazoans to both control tissue architecture and enable cancer prevention.

The study of cell polarity and how it helped generate multicellularity in the basal metazoans represents a rich opportunity to identify the original mechanisms that establish and maintain the organisation of tissues. Because of the high conservation in gene function between basal metazoans and Bilateria, these studies are also likely to provide broader insights into regenerative medicine and human cancer. Furthermore, identification of any divergent cell polarity mechanisms between basal metazoan and bilaterians will inform us as to the diversity and evolution of these core cellular mechanisms.
